# The experiences of parents of children living with disabilities at Lehlaba Protective Workshop in Sekhukhune district of Limpopo province

**DOI:** 10.4102/ajod.v8i0.528

**Published:** 2019-09-19

**Authors:** Brian Tigere, Jabulani C. Makhubele

**Affiliations:** 1 Department of Social Work, University of Limpopo, Sovenga, South Africa

**Keywords:** experiences, parents, children, disabilities, attitudes

## Abstract

**Background:**

Parents of children with disabilities have faced difficulties in looking after their children, be it socially, economically and financially. Parents in rural areas are mainly left with a huge burden, as there is a lack of services and support from both the state and non-governmental organisations. Parents in Sekhukhune district, a rural area in Limpopo province of South Africa, face challenges in raising their disabled children related to lack of resources and lack of services at their disposal.

**Objectives:**

This study focuses on the experiences and life circumstances faced by parents of children living with different types of disabilities at Lehlaba Protective Workshop in Sekhukhune district of Limpopo province, South Africa.

**Method:**

The study consisted of 14 participants who are parents of children living with disabilities. An interview guide with a set of questions was utilised to gather data. Thematic analysis was used to analyse the data and themes that emerged were grouped together.

**Results:**

Themes that emerged from the data showed that most of the participants had varying understandings on the causes of disabilities to their children. The participants also were of the view that a ‘cure’ for disability was available medically, spiritually or through traditional African medicine. The study also brought the notion of absent fathers, as most men do not want to be associated with children who are disabled. Stigmatisation of the parents was also a theme that the study revealed. The parents are subjected to name labelling as they are viewed to be practising witchcraft or to be paying for their sins they committed.

**Conclusion:**

Parents of children with disabilities are in their own battle in raising their children. There is a lack of support structures available for parents of children living with disabilities. There is a lack of legislation available for protecting and promoting the rights of children with disabilities. The researchers concluded that raising a child with a disability is expensive, time-consuming and straining.

## Introduction

Disability is the consequence of an impairment that may be physical, cognitive, mental, sensory, emotional, developmental or some combination of these, and it may be present from birth or may occur during a person’s lifetime (World Health Organization 2011). As a result, disability can affect not only those who are disabled but also those who are the primary caregivers. Raising a child with a disability is a challenge to most parents. In other words, primary caregivers, more especially those in rural settings, require assistance from all stakeholders, be it government or the private sector. The experiences faced by the parents, either negative or positive, have made an impact on the well-being of children living with disabilities. Primary caregivers include biological parents of the children or legal parents who include adoptive or foster parents.

For most parents, the birth of their first child is a major transition in life that involves the new challenge of caring for an infant, and major changes in lifestyle and sense of identity (Gupta 2011:74). The birth of a child with a disability, or the discovery that a child has a disability, can have profound effects on the parents or the family (Brown, Goodman & Kupper 2014). With the arrival of each new child in the family, further changes will take place. When a child is diagnosed with a disability, the experience of parenthood is affected, and expectations with regard to the child and the future may have to be revised.

Like all other children, a disabled child is born into a family and remains a lifelong family member. Among all the social institutions, for example the church, the school and the community, family is the most significant and is universally regarded as exerting the most influence on the child’s development as it provides care, love, support, protection, guidance and direction to children (Monk & Wee 2008:104). The way parents treat a disabled child is a reflection of attitudes they have towards disability.

South Africa has one of the best policies for children with disabilities, notably being a signatory to the United Nations Convention on the Rights of the Child (UNCRC) in 1995 and the United Nations Convention on the Rights of Persons with Disabilities (UNCRPD) in 2007. This has however failed to change the lives and living conditions of children with disabilities in South Africa in general and Limpopo province in particular. In addition, significant knowledge gaps remain with regard to the situation of the parents of children living with disabilities, their families and the underlying causes of their situations.

This study explored the experiences of parents of children living with disabilities at Lehlaba Protective Workshop in Sekhukhune district of Limpopo province. Lehlaba Protective Workshop is a rural community-based centre which provides day care stimulation services and protected work to children and adults with different kinds of disability. This is an important area of research to explore because available literature focuses on children with disabilities rather than their parents or guardians. All the problems faced by the children have an effect on their primary caregivers who are their parents and guardians. Therefore, the researchers saw it fit to explore the topic so as to highlight the journey faced by parents in raising their children living with disabilities.

## Background information

In this study, the term ‘disability’ means a physical, mental or sensory impairment, whether permanent or temporary, which limits the capacity to perform one or more essential activities of daily life, and this can be caused or aggravated by the economic and social environment (Schulze 2010). A parent is the caretaker of a child. Physical disability is any impairment that limits the physical function of limbs and loss of motor ability (Anastasiou 2013). According to Crow (2010:77), the term ‘intellectual disability’ is used when a person has certain limitations in mental functioning and in skills such as communicating, taking care of himself or herself and social skills. Mitra, Posarac and Vick (2011) stated that there is no consensus on a definition and measurement of the controversial and complex phenomenon of disability. Different conceptual models have been developed for definitional purposes, including the charity, medical, economic and social models (Altman 2001:105). The charity model views persons with disabilities as elements of pity and, therefore, to be helped by welfare approaches. The medical model considers disability as a problem of the individual directly caused by a disease, an injury or some health condition and requiring medical care in the form of treatment and rehabilitation. Individuals with any impairment are considered disabled, where ‘impairment’ is used for their condition, irrespective of whether the individuals experience limitations in their activities. The medical model is often opposed to the social model, which views disability purely as a social construct where the problems of the disabled are either caused or exacerbated by the society in which they live (Mitra et al. 2011).

The *Social Assistance Act* No. 13 of 2004 of South Africa defines a person with a disability as a person who is, owing to a physical or mental disability, unfit to obtain by virtue of any service, employment or profession the means needed to enable him or her to provide for his or her maintenance (Government Gazette 2004).

In general, research of children with disabilities in South Africa has been conducted. However, experiences of the parents of children with disabilities in rural areas, especially in Limpopo province, have never been well documented. Research has however been conducted with parents of children living with disabilities notably in developing countries, such as Zimbabwe and India.

In most parts of Southern Africa including Zimbabwe, children born with albinism used to be killed immediately after birth. The parents had to endure the pain of watching their children being killed as they did not have power to stop the killings. Furthermore, people with disabilities in Zimbabwe were marginalised and treated as if they were not capable of functioning on their own. Disability was equated with inability. In most parts of Africa, including Zimbabwe, disability is viewed as either a form of punishment or as a curse by angry ancestral spirits (Chimedza & Peters 2006:425).

In Zimbabwe and other African nations, the family has been responsible for taking care of its disabled members. However, pressures from urbanisation and changing patterns of employment leading to urban migration have resulted in the breakdown of the extended family system (Chimedza & Peters 2006:428). Because of more limited family support, children with disabilities have been locked in houses and placed in institutions and are generally seen as burdensome (Chimedza & Peters 2006). Tolerance of people with disabilities has also tended to diminish sharply during periods of economic hardship (Choruma 2007). This has left the burden on the parents who have to bear the brunt of looking after the children with disabilities.

Parents of children with disabilities in India face the challenge of poverty associated with disability. A large number of children with disabilities live in families with income significantly below the poverty level. According to Miles (2000:613), ‘while disability causes poverty, it is also possible that in a country like India, poverty causes disability’. The combination of poverty and disability results in a condition of simultaneous deprivation.

The attitudes of the non-disabled are proving to be a major barrier in the social integration of children with disabilities. The more severe and visible the deformity is, the greater is the fear of contagion, and hence the attitudes of aversion and segregation towards the crippled (Miles 2000:606). Such attitudes reinforced by religious institutions may militate against any attempts to include students with disabilities into regular schools. Hindus (who constitute 85% of the total population in India) believe that disability is a consequence of misdeeds performed in the previous life. Many Hindu religious institutions and temple trusts, therefore, do not think a part of their duty is to help children with disabilities because they consider disability to be the result of a person’s misdeeds in his or her previous life. Parents of the children are left to suffer emotionally to this hostile society (Hastings & Beck 2004). In India, disability is still viewed in terms of a ‘tragedy’ with a ‘better dead than disabled’ approach, the idea being that it is not possible for disabled people to be happy or enjoy a good quality of life (Padencheri & Russell 2004:134). Cultural beliefs about disability play an important role in determining the way in which the family perceives disability and the kind of measures it takes for prevention, treatment and rehabilitation. In rural India, parental expectations for their disabled child are mostly negative and unrealistic (Gupta & Singhal 2014:28).

## Objectives

The first objective of this study was to describe and explore the experiences faced by parents of children with disabilities at Lehlaba Protective Workshop in Sekhukhune district of Limpopo province, South Africa. The second objective was to identify resources available for parents of children with disabilities.

## Research method and design

To achieve the objectives of the research, the researchers employed a qualitative methodology. According to Babbie, Mouton and Strydom (2011), social research can be conducted for explanatory, descriptive or exploratory purposes. Explanatory studies aim to provide causal explanation of phenomena. An exploratory approach was appropriate for this research because the researchers’ purpose was to explore the experiences of parents of children living with disabilities at Lehlaba Protective Workshop. As most studies have focused on children living with disabilities as subjects, there is little information available about parents with regard to their experiences in taking care of their children, especially in a rural setting.

Purposive sampling was used to collect data from 14 participants. Out of these, nine were women and five were men. Twelve of the participants were biological parents, being the father or the mother, while two were legal guardians, one being an aunt and another being a sister. The legal guidance was facilitated through the *Children’s Act* of 2008 (Foster Care). The population of the study comprised parents of children with disabilities attending Lehlaba Protective Workshop of Sekhukhune district of Limpopo province. Lehlaba Protective Workshop is a community-based disability centre that offers stimulation activities to children with disabilities. The biological profile of the participants is presented in Table 1. For ethical reasons, pseudonyms were used to protect participants’ identifying details.

**TABLE 1:** Biological profile of the study participants

Name	Age (year)	Sex	Marital status	Occupation	Relationship	Age of child (year)	Care dependency grant status
Parent A	33	Male	Married	Electrician	Father	8	Yes
Parent B	29	Male	Married	Boilermaker	Father	11	Yes
Parent C	42	Female	Single	Unemployed	Mother	13	Yes
Parent D	37	Female	Single	Unemployed	Mother	8	Yes
Parent E	42	Female	Single	Unemployed	Mother	10	Yes
Parent F	29	Male	Married	Motor Mechanic	Father	10	Yes
Parent G	38	Male	Married	Builder	Father	8	Yes
Parent H	43	Male	Married	Mine worker	Father	9	Yes
Parent I	34	Female	Single	Unemployed	Mother	7	Yes
Parent J	37	Female	Single	Unemployed	Mother	9	Yes
Parent K	29	Female	Married	Secretary	Mother	11	Yes
Parent L	38	Female	Single	Unemployed	Mother	10	Yes
Parent M	36	Female	Single	Unemployed	Auntie	10	Yes
Parent N	24	Female	Single	Receptionist	Sister	8	Yes

The age of the participants ranged from 24 to 43 years. A total number of 14 people participated in the study. Seven of the parents were unemployed, while the other seven were employed. Six of the respondents were married, while eight were single. Of importance to note is that seven of the single parents are unemployed.

The interview is a social relationship designed to exchange information between the participant and the researcher (De Voset al. 2011). The study utilised in-depth interviews for individual participants and these were particularly useful for this study because they encouraged the participants to respond freely and for the researchers to probe for more information. The parents of children with disabilities were interviewed in offices at Lehlaba Protective Workshop so as to make them relax and avoid nervousness among them.

Data analysis is a process of bringing order, structure and meaning to the mass of collected data (Padgett 2016:68). Qualitative data from in-depth interviews and observations were analysed according to themes that emerged during brief direct observations and from the discussions with the participants.

The researchers transcribed all the interviews, a process that entailed listening to each interview and typing it. The researchers read the transcripts several times to become familiar with the information and during this process they made memos and looked at the similarities and differences emerging from the data. The researchers also made marginal notes on the transcripts, which helped in the initial process of exploring and analysing the data.

The researchers went on to classify information by grouping together similar responses, a process described by Rubin and Babbie (2011) as taking apart text or qualitative information and looking for categories, themes or dimensions of information. This process is called coding. Coding is the process of combing the data for themes, ideas and categories and then marking similar passages of text with a code label so that they can easily be retrieved at a later stage for further comparison and analysis (Padgett 2016). In this study, data were coded using themes that emerged from the discussions.

The researchers identified themes, categories and sub-categories that fell in line with the main research objectives. The researchers continuously examined the information, comparing and categorising the data, and breaking the categories down into fewer and more inclusive terms. This manual process enabled him to analyse the results as well as to develop an initial awareness of issues that came up in the interviews. The themes that emerged from the data analysis are presented in the ‘Results’ section of this research report.

## Ethical considerations

Permission to conduct the study was obtained from Research and Ethics Committee of the University of Limpopo (project number: TREC/133/2016:PG). Permission was also sought from the parents of children with disabilities and they consented for their participation through the signing of consent forms. The participants were also informed that participation was voluntary and that they could withdraw from the study voluntarily without any consequences. To ensure anonymity in the study, steps were taken to protect the identity of the participants by neither giving their actual names when presenting research results nor including any identifying details that could have revealed their identity, such as workplace, personal characteristics and occupation. The interview schedule also did not carry the actual names of the participants but rather tags in the form of numerical numbers and alphabetical letters. Confidentiality was also preserved by conducting the interviews in private rooms. Data collected from the study were kept confidential in a safe locker at the Department of Social Work offices.

### Trustworthiness

Trustworthiness in research is demonstration that the evidence for the results reported is sound and when the argument made based on the results is strong (Denzin & Lincoln 2008). Four criteria to ensure valid interpretation of data were used in the study: truth value (credibility), applicability, consistency and neutrality.

### Credibility

In addressing credibility, investigators attempt to demonstrate that a true picture of the phenomenon under scrutiny is being presented (Shenton 2004:66). Credibility was ensured by using a qualitative approach to determine the experiences of parents of children with disabilities. The use of a purposive sample by the researcher ensured that the responses obtained are credible. The selection criteria used were that the respondents should be biological or foster parents of children living with disabilities. Open-ended questions ensured that participants provide their opinions without the influence of the researcher. The interview also allowed the researcher to note non-verbal communication. Credibility in the study was also ensured through investigator triangulation. A research assistant who was part of the team coded, analysed and made interpretations of the data from the recordings and notes that were written down. Same themes, results and interpretations were yielded.

### Transferability

Transferability relates to the extent to which the findings of one study can be applied to other situations (Rubin & Babbie 2011). In this regard, background data which include detailed information of the field area under study, which is Sekhukhune district of Limpopo province, have been given.

### Dependability

Dependability entails that researchers should at least strive to enable a future investigator to repeat the study (Shenton 2004). In this study, the researchers described in detail the research design, which is a framework for the collection and analysis of data. The researchers mentioned the method of data collection, which included in-depth interviews. To achieve this, the data collection instrument of an interview schedule was mentioned. Such detailed description of the methods in the research provides information on how repeatable the study can be.

### Conformability

Conformability refers to the fact that researchers must take steps to demonstrate that findings emerge from the data and not their own predispositions (Shenton 2004). Conformability was assured in this study by providing a detailed and informative research methodology. To achieve conformability, detailed records of data collected were kept for this study for peer review or outside audit if required.

## Findings of the study

Three main themes that emanated from the study were:

- beliefs about disability
- resources for children with disabilities
- psychosocial experiences.

### Cultural beliefs about disability

Cultural beliefs define who people are, how they interact with the world and how they behave in certain situations and can be considered a combination of religious beliefs, socially accepted norms and traditions. Different cultural groups have different perceptions of the causes of disability (Omu & Reynolds 2012:124). This section will focus on the participants’ understanding of the causes of disability, cure for disability and their general understanding of the term ‘disability’.

### Parents’ beliefs on the causes of disability

The parents of children with disabilities had varied explanations on the causes of disability among their children. Some parents voiced out that witchcraft (use of ‘black magic’) was the cause of their children’s disabilities. Some echoed out that problems at birth (40%) were the cause, while some other attributed their children’s disabilities to motor vehicle accidents (30%).

These findings on causes of disabilities were from the responses of the parents:

‘I suspect witchcraft because during my pregnancy one of my neighbours who I owed money told me she was going to fix me. I had prolonged labour and I suspect her for bewitching me.’ (Parent J)

‘My husband was having another wife before she married me. The former wife told me she was going to curse me. It became a reality when I gave birth to my epileptic disabled child.’ (Parent L)

The above responses demonstrate that some of the parents believe that their children’s disabilities are a result of witchcraft.

Some of the parents however believed medical reasons to be the causal effect of disability in their children.

‘I got into labour when I was home and alone. My neighbours who came for assistance were not skilled in any way to assist me. When the ambulance came I had already delivered the baby. I was told by the nurses that my baby did not have enough oxygen during delivery.’ (Parent I)

This parent believes that the disability of her children was caused by a lack of qualified medical personnel to assist her in the safe delivery of her child.

‘My child had Polio. I did not act quickly since I realized it late that my child was having difficulty in crawling and walking. When I went to the hospital for treatment it was too late.’ (Parent K)

The parent in the above response is acknowledging the fact that her son’s disability was caused by a medical condition, which is polio.

One participant responded by saying a motor vehicle accident caused his son to be disabled and he does not believe in witchcraft.

‘My son was involved in a minibus taxi accident in 2011 when he was 5 years old when coming from holiday in Pretoria. He sustained head injuries which caused brain damage. The doctors also said his spinal cord was disturbed.’ (Parent H)

The above responses point out that the child’s disability was caused by a motor vehicle accident.

The participants thus had different views on the causes of disability to their children. Some were of the opinion that witchcraft paid a part, while others noted medical reasons.

### Parents’ beliefs on a cure for disability

Parents look for a cure for disability of their children in the early years. Parents spent a great amount of money in trying both the Western and African medicine for a cure of their children’s disability.

The parents had different views and responses when it came to a cure of their children.

‘I believe one day the medicals will come up with a medical solution for my intellectually disabled son. I do get a drug called Epilim every month at Dilokong Hospital. That drug helps my son to desist from aggression. I believe one day they will come up with a permanent drug to end his disability. Just look at ART Therapy (anti-retroviral therapy). Now they are going towards finding a drug to treat HIV/AIDS completely.’ (Parent N)

This guardian is of the belief that his son will be cured completely of his disability one day.

‘I have accepted that my son will spend his whole life in a wheelchair. I have tried by going to most Churches even attending crusades of foreign preachers in Gauteng but nothing has changed on the condition of my child. I do now believe that miracles happened during Jesus times unless the Son of God resurrects again. It is a hard decision to take but I have to accept the reality that my son is disabled for the rest of his life.’ (Parent H)

‘I have been to prophets who come to Gauteng from other African countries hoping that my child will be cured. I just have to admit that it has failed and I have to accept and live with the reality.’ (Parent A)

‘I have gone from one traditional healer to the other, both locally and regionally to Mozambique and Zimbabwe but no ‘Sangoma’ has cured the disability of my child.’ (Parent F)

It can be summed up that all the parents of children with disabilities searched for a cure of disability for their children. Some are still hoping to find the cure, while some have accepted the disability of their children is lifelong.

### Parents’ understanding of disability

Parents’ understanding about their children’s disability was surprising. All the parents did not understand their children’s disabilities well. They provided a general view about their understanding of disability.

‘I only know that my child has got a condition called cerebral palsy but I do not know what it means. After giving birth, no one (medical staff) bothered to inform me what I need to do in order to take care of my child. The experiences I am having are teaching and guiding me. It is like I am learning from the experiences I have.’ (Parent J)

‘My child is autistic. He is 8 years. No one has ever informed me about handling a person with autism. Sometimes you are not sure if you are doing the right thing. It is just scary not to know and understand your child.’ (Parent L)

‘Her abilities and limitations is what I do not know. This led me to underestimate her on her abilities sometimes. I was of the opinion that we should as a family do everything for her since she is on a wheelchair. One day she surprised us by saying she can make her bed alone. I felt guilty because I did not understand her disability.’ (Parent M)

These three participants’ expressions unearthed that they are not sure of their children’s disabilities. Their understanding of disabilities is limited. This leads to unintentional harm to their children with disabilities.

### Resources for children with disabilities

#### Assistive devices

Assistive devices include tools or equipment designed for the mobility of persons with disabilities. Assistive devices can be in the form of wheelchairs (standard or motorised), crutches, walking frames and eating frames (WHO 2011). Responses from the participants were as follows:

‘My child uses a specialised wheelchair known as the Buggy. This buggy is more expensive than the standard wheelchair. This specialized wheelchair offers rehabilitation and avoids secondary disabilities. Since it is expensive, I have to use a standard wheelchair and sometimes a wheelbarrow which is not suitable for my child and can cause secondary disabilities. I cannot afford the specialized wheelchair since I do not have any reliable source of income.’ (Parent E)

From the above discussion, it can be inferred that the parents are struggling to purchase assistive devices. Some of the parents resort to using wheelbarrows as wheelchairs are scarce. This has got a negative effect as it causes secondary disabilities.

### Professionals

Allied health professionals are paramount in the rehabilitation and upkeep of children with disabilities. They include social workers, psychologists, occupational therapists, physiotherapists and speech therapists.

A guardian (foster parent) of a child with epilepsy reiterated that:

‘We do not have even a social worker. If we want the professionals, we go to Dilokong Hospital which is roughly 55 kilometres away. Imagine travelling with a child with a disability for such long distances. And she really needs the services of a physiotherapist to rehabilitate her bodily movements.’ (Parent I)

The physically disabled require constant exercises from different therapists such as physiotherapists to avoid secondary disabilities.

Parent I (single unemployed mother) asserted that, ‘my child has never received any help from a physiotherapist or speech therapist as my child has problems with mobility and speech’.

It can thus be noted that allied health professionals in the study area are not accessible to children with disabilities because of the distance.

### Psychosocial experiences

#### The social life of the family

Having a child with a disability tremendously affects everyone in the family. This includes the child’s immediate family members, who can be brothers or sisters. Parents of children with disabilities brought to attention that isolation is now common in their life, as they no longer have a life other than looking after their disabled child. The following responses are in support of the above statements:

‘I always have a very busy day at home. I have to make sure that everything is in place.’ (Parent J)

‘My other two children sometimes complain that I do not give them attention since most of the time I am always looking after their disabled young brother.’ (Parent K)

It should be noted that parents’ isolation is more evident in the mothers because the functions inherent to being a mother, housewife and permanent caregiver are time-consuming. The same does not apply to the fathers who are mostly breadwinners and at work most of the time.

Leisure activities for disabled people require an additional effort from parents, as society still does not recognise leisure as an essential right of disabled citizens (Duncan, Sherry & Watson 2011:34). Responses from the participants supported this notion that parents do not go out for leisure activities with their disabled children.

‘I do not go out with him since it is costly as I have to hire a private car.’ (Parent C)

‘He spends the weekend at home, lying down or sitting in bed.’ (Parent H)

### Distress on time

Most parents spend most of their time taking care of their children living with disabilities. Some of the responsibilities of the parents include bathing, preparing special dietary meals, feeding, toiletry duties and changing diapers or nappies. There was a sense of frustration in the amount of time devoted to caring for the children with disabilities. One parent was of the view that only death of the child is an end to the time spent on caring for the child.

‘Actually all my time is spent on looking after her since I have to do almost everything for him: bathing, feeding, and changing clothes. Almost everything. It means I do not have time for other things.’ (Parent K)

‘All my time is now bound on taking care of my child with cerebral palsy, I have to do any little task for her as there will not be anyone to do it for her. I am scared to say it will be until God’s will but that is the reality.’ (Parent C)

### Fears of another child

All the biological parents voiced their fears of having another child. Both men and women were of the view that they will never try to conceive again as they are scared of having a second child with a disability. This is mainly because of the demands which are involved in taking care of a disabled child.

‘I am facing a lot of untold challenges in raising this child. This makes me reluctant to have another child. What if I conceive a disabled child again?’ (Parent E)

‘This one who is disabled is the last born in a family of three children. I do not want to risk having another child who might turn out to be disabled.’ (Parent H)

‘This is my first and last child. I do not want to end up having two disabled children. I have a disabled child now, what can stop me from conceiving another disabled child.’ (Parent I)

## Discussion of findings

The study findings have noted negative aspects which are mainly encountered by parents of children living with disabilities at Lehlaba Protective Workshop in Sekhukhune district of Limpopo province. The study has revealed that parents of children with disabilities have varying beliefs on the causes of their children with disabilities. The study noted that witchcraft is mainly to blame for disabilities of their children. These sentiments are echoed by Omu and Reymonds (2012:125), who are of the opinion that most African parents believe witchcraft as a cause of disability to their children. Medical reasons were also noted in the study as having disabled their children. In support, Anastasiou (2013:445) is of the same view that disability is a medical condition which requires medical means to resolve.

Findings from the study have highlighted that parents usually looked for a cure of their children’s disabilities. Most of the parents looked for medical solutions, while others looked for divine interventions through religious means. Some parents went a step further through the services of traditional healers and ‘sangomas’. In support of these claims, Dillenburger and McKerr (2011:37) noted that parents usually spend a fortune in trying to find a cure and solution to their children’s disability. Dowling and Dolan (2001:31) also share the same sentiments in that most parents take much time looking for a cure and in doing that take time in accepting their child as disabled. The findings of the study have shown that parents are not usually aware of their children’s disabilities and this has negative effects on their upbringing. Gupta and Singhal (2014:34) also share the same sentiments in that in India parents of children with disabilities are not quite sure of the disability of their children. They just generalise which results in unintentional harm and secondary disabilities. Results from the study have shown that resources for children with disabilities are problematic and scarce. Assistive devices, for example wheelchairs, walking and eating frames, are always in short supply. Wheelbarrows are used as an alternative, which can cause secondary disabilities. In support, Visagie, Scheffler and Schneider (2013) opined that wheelchairs in rural areas are limited mainly because of the long process of application as well as lack of adequate funding. Glumac et al. (2009:168) also noted that assistive technologies for rural children hinder their rehabilitation and progress in their livelihoods. Services from professionals are critical in the field of disability rehabilitation. The study revealed that professionals such as audiologists, physiotherapists and occupational therapists are in short supply. This hinders their rehabilitation progress. In support, Chitereka (2010:87) highlights that professionals who are pivotal for rehabilitations are in short supply in most rural areas.

Family disruptions have been seen in the study as negative implications of having a child with a disability. More attention is given to the child with special needs, and neglect takes centre stage from other children within the household. The study findings in this regard relate to Legg and Penn (2013:139), who are of the view that the presence of a disabled child affects each sibling individually, as well as the relationships between siblings. In short, the social life of the family is disrupted. Having a disabled child also puts a strain on time of the parent or guardian. Gona et al. (2011:181) also share the same sentiments in that for caregivers, mostly parents of children with disabilities, life is put on hold as they have to be available always for their children with special needs. A significant amount of time is spent on caregiving activities, such as feeding and changing diapers. Thus, it can be concluded that parents of children living with disabilities are left without any time to do other activities, such as employment or leisure.

Problems associated with raising a disabled child have left many parents scared to have children altogether. The study findings noted that the burden associated with raising a disabled child made parents uncomfortable to have children. These sentiments are supported by Hill and Rose (2009:972) who postulated that stress of raising a child with disabilities is associated with fears of trying to have another child altogether. Therefore, challenges associated with raising disabled children deter parents from trying to have children.

## Recommendations

The findings and literature review from this study have prompted the following strategic recommendations.

### Care Dependency Grant

The Care Dependency Grant (CDG) needs to be awarded according to the nature of disability of the children. For instance, children with severe disabilities like cerebral palsy need to be awarded more financial resources as it is costly to look after those children. The Department of Development through its distributing agency, South African Social Security Agency, needs to conduct a feasible study on the impact of CDG in relation to the nature of disabilities.

### Healthcare and rehabilitation

The Department of Health of Limpopo province needs to come up with mobile clinics such that their services reach children with disabilities. The nearest health facility is approximately 55 km away and it makes it difficult for parents to transport their children for medical services. Therapists (e.g. occupational, speech and physiotherapists) need to conduct fieldwork visits to centres for children with disabilities to foster community-based rehabilitation.

### Legislation

Legislations available for children with disabilities include the Constitution of South Africa and the *Social Security Act*. There is a need for a *Comprehensive Disability Act* like other developed countries such that rights of children with disabilities can be advanced.

### Support groups

Parents endure a great deal of emotional stress and burn-out when taking care of their children with disabilities. The Department of Social Development needs to vigorously implement the formation of support groups for parents of children living with disabilities. These support groups will be instrumental in providing psychosocial support to parents of children living with disabilities. Sharing ideas and meeting each other’s emotional needs help to remove the burden on the shoulders of the parents. The parents also need to start support groups through social media platforms such as WhatsApp and Facebook groups. This is less expensive and less time-consuming.

## Conclusion

The research study has made it clear that parents face negative experiences when raising their disabled children. Parents demonstrated different views regarding the causes of their children’s disabilities. Some were of the view that witchcraft was the cause of disabling their children. This finding supports those of Mazibuko (2011), who urges that disability in the African context is mostly understood through African means, such as witchcraft and bad spirits. In support of this, Gupta (2011) highlighted that most parents in developing countries do not have a clear understanding of their children’s disabilities.

The researchers can conclude that parents of children with disabilities in the study area are in their own battle in raising their children. There are no available resources, both from the government and the private sector. It could also be concluded that there are varying conceptions about the causes of disabilities among children. Some of the participants felt that they were left alone in the struggle of looking after their disabled children. Some felt that they even do not have time for other activities, such as leisure and even employment. Thus, in brief, it could be said that raising a child with a disability is time-consuming and straining.
